# Supplementary figures and images for: Morphological and Physiological Changes of Broussonetia papyrifera Seedlings in Cadmium Contaminated Soil
Source: Plants (Basel). 2020 Dec 3;9(12):1698. doi: 10.3390/plants9121698 (PMC7761668; doi:10.3390/plants9121698)

5.07 mg/kg

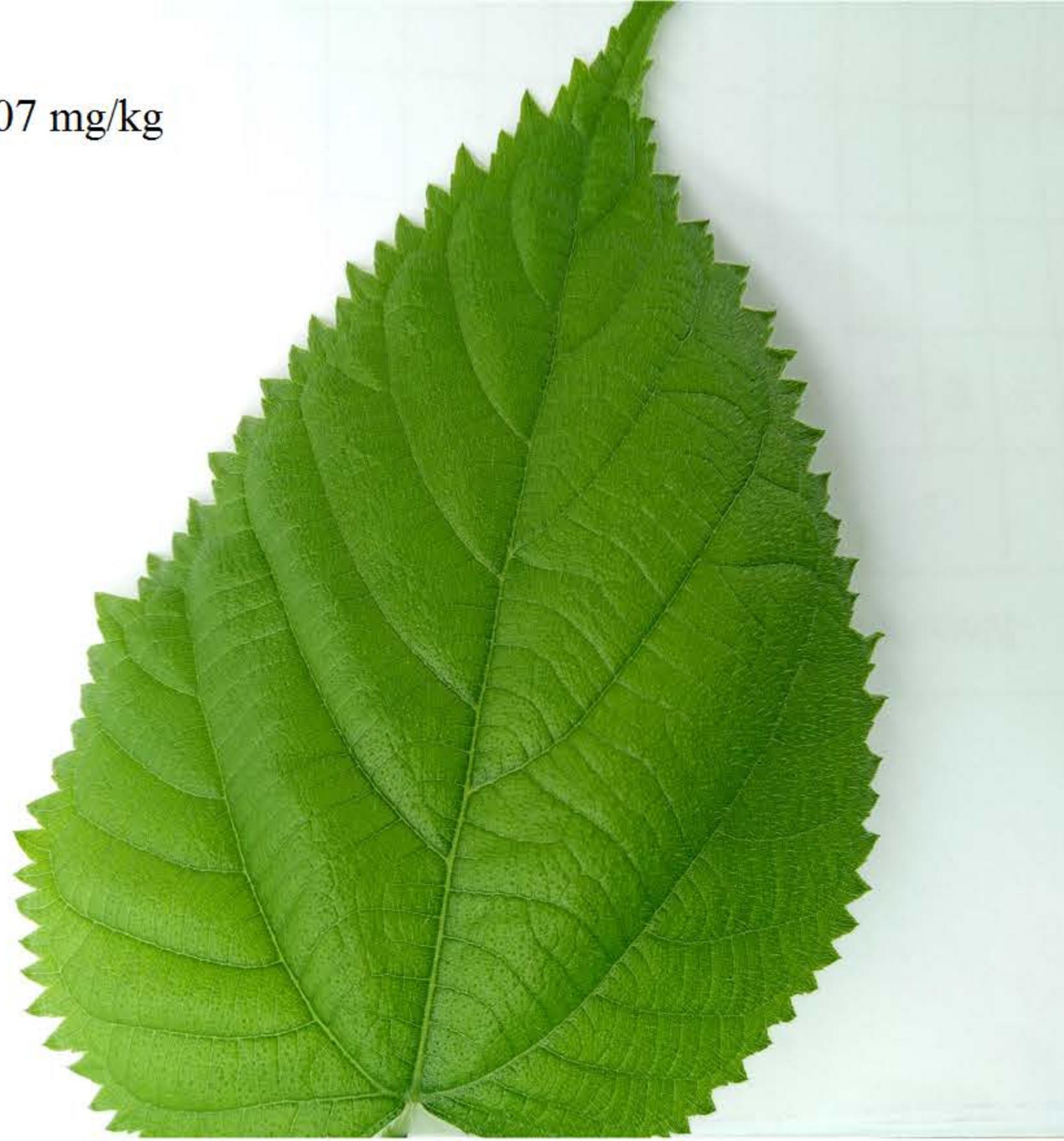

5.71 mg/kg

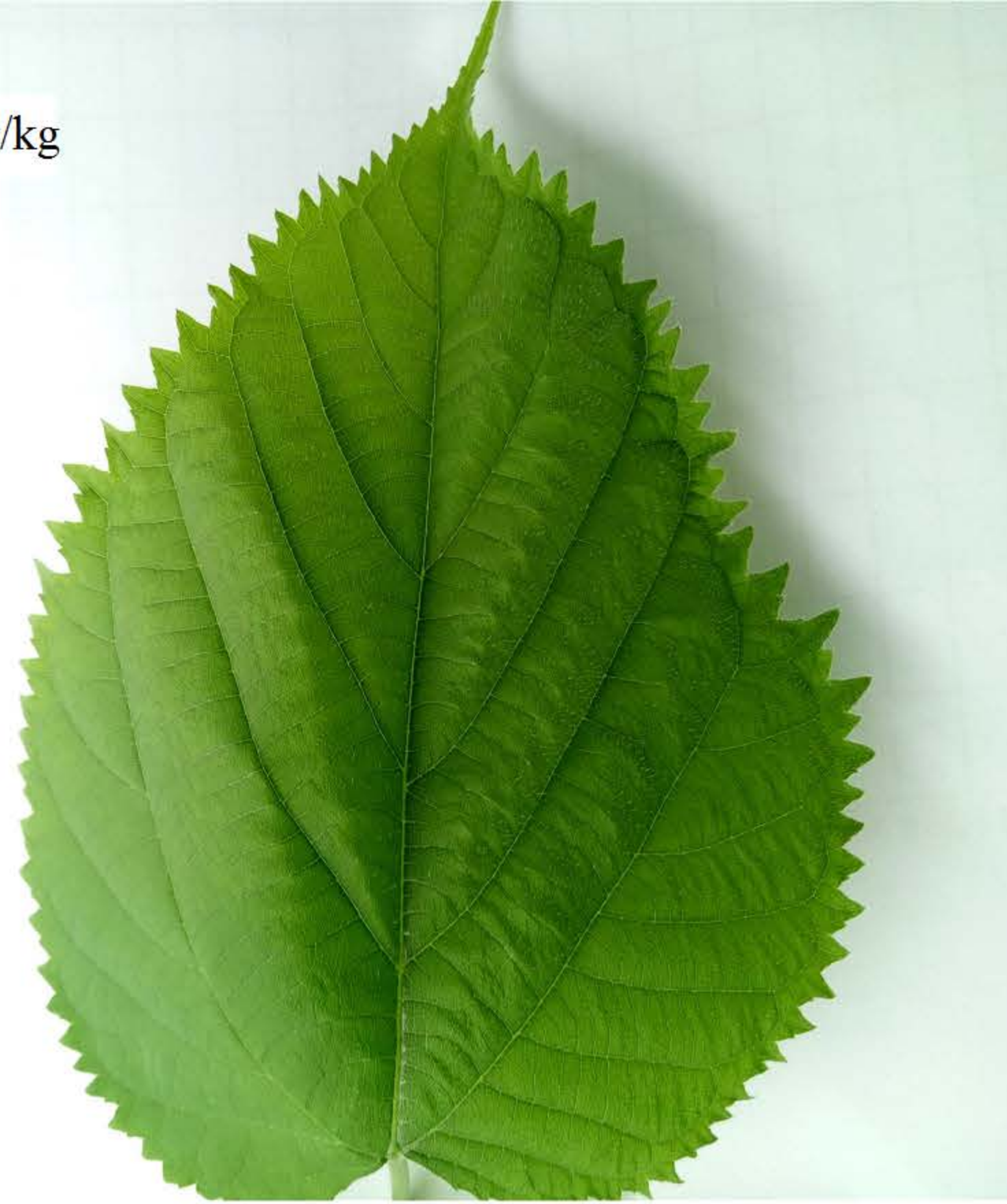

8.28 mg/kg

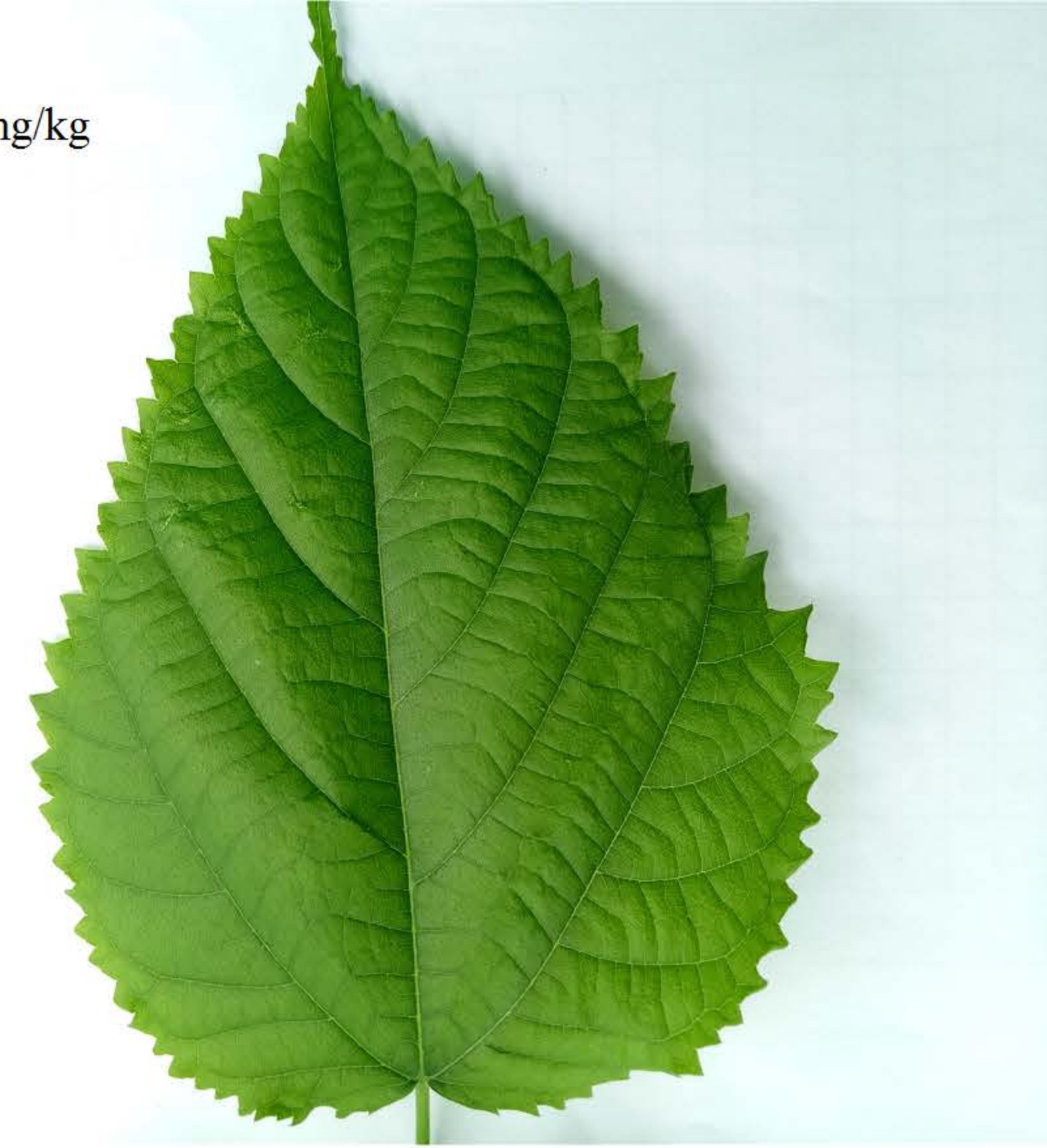

11.49 mg/kg

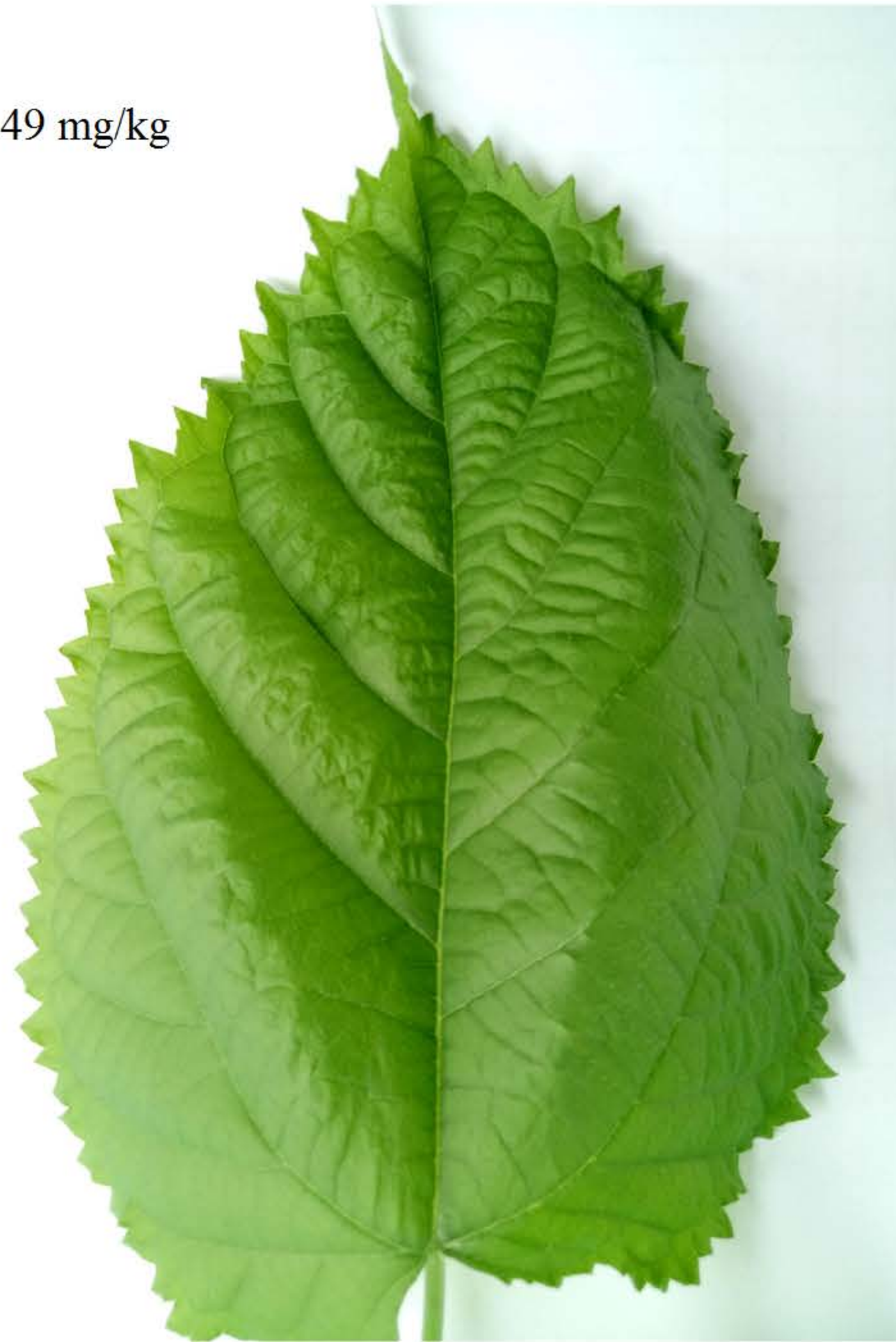

14.71 mg/kg

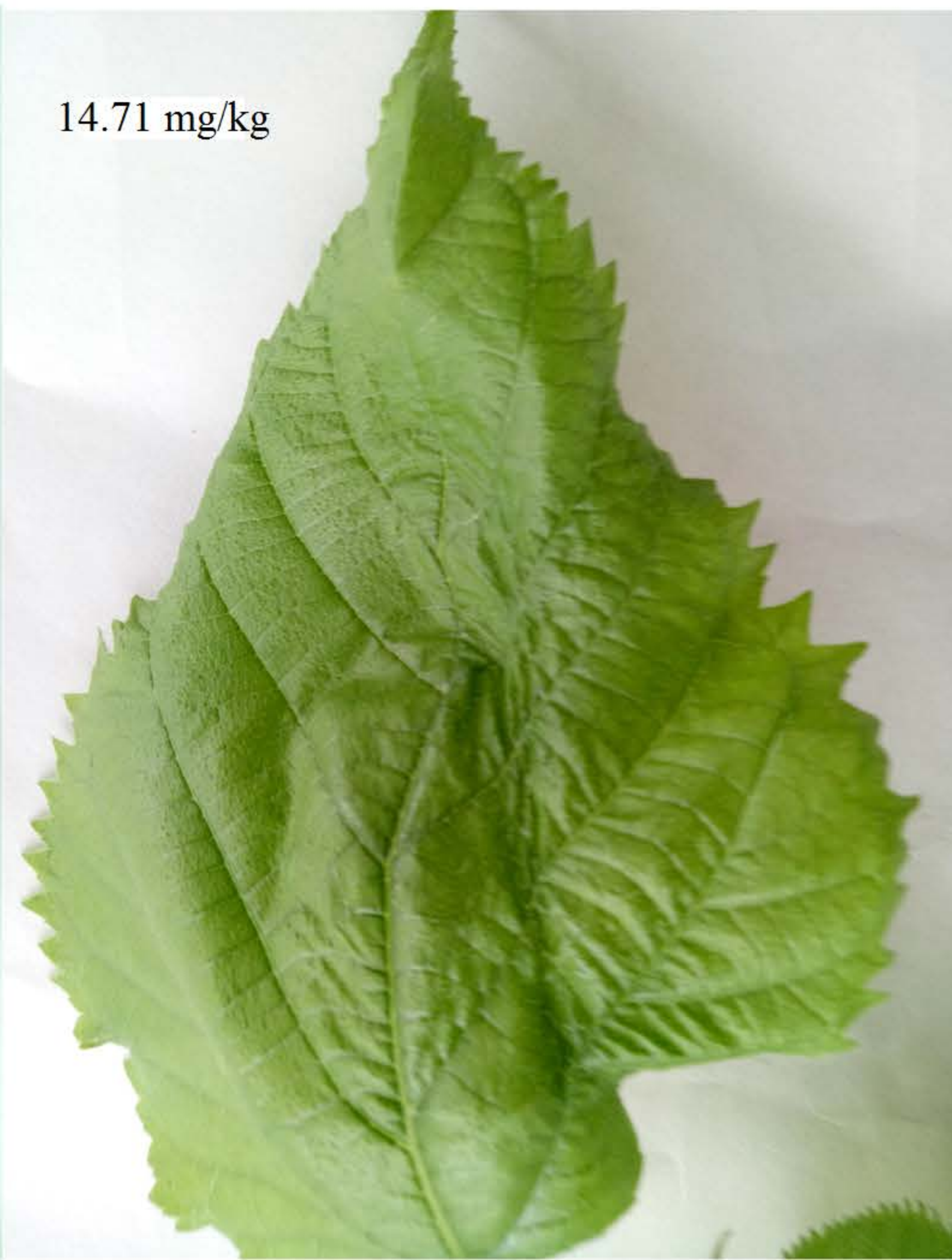

Supplement: Supplementary file 1 [file plants-09-01698-s001.pdf]
